# First report of tuberculosis in a cat from Italy caused by *Mycobacterium africanum*, lineage 6: genomic characterization and phylogenetic analysis

**DOI:** 10.3389/fmicb.2025.1633110

**Published:** 2025-09-01

**Authors:** Patricia Alba, Andrea Caprioli, Cristiano Cocumelli, Claudia Eleni, Valentina Galietta, Angelo Giacomi, Luigi Sorbara, Fiorentino Stravino, Fabiola Feltrin, Roberta Amoruso, Angela Ianzano, Francesco Ceccaroni, Mario Frega, Virginia Carfora, Alessia Franco, Antonio Battisti

**Affiliations:** ^1^Department of General Diagnostics, Istituto Zooprofilattico Sperimentale del Lazio e della Toscana “M. Aleandri”, Rome, Italy; ^2^Veterinary Practitioner, Rome, Italy; ^3^Azienda Sanitaria Roma 1, Servizi Veterinari, Rome, Italy

**Keywords:** *Mycobacterium africanum*, tuberculosis, genomics, antimicrobial resistance, zoonosis, cat, Italy, whole-genome sequencing (WGS)

## Abstract

**Introduction:**

Tuberculosis in humans is mainly caused by two closely related bacteria within the *Mycobacterium tuberculosis* complex (MTBC), which are *Mycobacterium tuberculosis* and *Mycobacterium africanum*. *M. tuberculosis* is widely spread, while *M. africanum* is more ecologically restricted to Africa.

**Methods and results:**

In 2023, we examined a skin biopsy from a 3-year-old female domestic cat with multifocal nodular cutaneous lesions and respiratory problems. The animal was an indoor cat kept in Rome, reportedly taken in as a stray kitten from a village in southern Italy (Central Calabria Region). Skin histology with Ziehl–Neelsen staining was consistent with suspected mycobacteriosis. Bacterial cultures for *Mycobacterium* spp. yielded an isolate, identified by polymerase chain reaction (PCR) as a *Mycobacterium tuberculosis* complex (MTBC). Whole-genome sequencing and bioinformatics further identified the isolate as *M. africanum* lineage 6, and phylogeny with 634 other MTBC genomes placed it within a West African cluster (mainly from Gambia) of the L6.1.2 sublineage. Resistome analysis indicated the presence of resistance genes intrinsic in *M. tuberculosis* and point mutations not associated with resistance. The cat died roughly 1 year later, most probably from systemic tuberculosis, but the owner did not request a necropsy.

**Discussion:**

This represents the first reported case of *M. africanum* infection in a carnivore and in a companion animal. The case history reports a stray kitten collected in an area of southern Italy, near the first migrant reception centers and croplands where workers coming from West Africa are often employed, consistent with our phylogenetic evidence.

## 1 Introduction

Mammalian tuberculosis (TB) is a chronic granulomatous disease that affects both animals and humans and is caused by bacteria within the *Mycobacterium tuberculosis* complex (MTBC). MTBC members belong to the family Mycobacteriaceae and are Gram-positive, acid-fast bacilli. The taxonomy of organisms in the MTBC is in a state of constant evolution (World Organisation for Animal Health, 2022). Recent genomic analyses suggest that all MTBC members belong to a single species, *M. tuberculosis*, with *Mycobacterium africanum*, *Mycobacterium bovis*, *Mycobacterium caprae*, *Mycobacterium microti*, and *Mycobacterium pinnipedii* considered heterotypic synonyms (variants) of *M. tuberculosis*, and *Mycobacterium canettii*, *Mycobacterium mungi*, and *Mycobacterium orygis* are recognized as strains of *M. tuberculosis* (World Organisation for Animal Health, 2022; Riojas et al., 2018). To retain linkage with historical nomenclature, the more widely recognized designations are commonly used instead of infra-subspecific designations (World Organisation for Animal Health, 2022). Hence, throughout the paper, we used the historical nomenclature (e.g., *M. africanum*) designations instead of *M. tuberculosis* var. *africanum* for ease of prior association.

All MTBC members share remarkable genomic similarity, with more than 99.95% nucleotide identity. Although all mammalian species are considered susceptible to tuberculosis, they vary considerably in their host tropism and ability to cause disease (Silva-Pereira et al., 2019). Single nucleotide polymorphisms (SNPs) and deletions of genomic regions ranging from 2 to 12.7 Kb, denominated “regions of difference (RDs),” allow for species differentiation (Brosch et al., 2002; Cousins et al., 2003); World lineage-wise classification of MTBC was also achieved using restriction fragment length polymorphism (RFLP) and PCR, such as mycobacterial interspersed repetitive units – variable number of tandem repeats (MIRU-VNTR) spoligotyping (Brosch et al., 2002).

*M. tuberculosis* and *M. africanum* are primarily human pathogens, but are also known to infect animals, and are considered pathogens of high Public-Health and One-Health relevance (Comín et al., 2021; World Organisation for Animal Health, 2022). *M. tuberculosis* is widely spread, while *M. africanum*, first described in 1968 from TB patients in Senegal (Castets et al., 1968), is more ecologically restricted to Africa and, in particular, West Africa, where it is responsible for almost half of all the TB cases (Comín et al., 2021; World Organisation for Animal Health, 2022; Silva et al., 2022). TB caused by *M. africanum* strains outside West Africa, although rare, has been described in several countries, mostly found in migrants from endemic areas (Yeboah-Manu et al., 2017; Comín et al., 2021; Silva et al., 2022).

Ten human-adapted lineages (L) belong to the MTBC. In particular, L1–L4 and L7 and L8 comprise *M. tuberculosis sensu stricto* (which majorly infects humans), while L5, L6, L9, and L10 consist of *M. africanum* (Blouin et al., 2012; Firdessa et al., 2013; Riojas et al., 2018; Gagneux, 2018; Ngabonziza et al., 2020; Coscolla et al., 2021; Balamurugan et al., 2022; Silva et al., 2022; Guyeux et al., 2024). L6 is known to be geographically restricted to West Africa, and L5 is known to have moved from West Africa to Central Africa (Coscolla et al., 2021; Balamurugan et al., 2022). In contrast, L9 belongs to a sister clade of L6, being placed between L6 and the animal-adapted lineages (Coscolla et al., 2021). Recently, a proposed L10 was also described, a sister lineage of L6 and L9 associated with Central Africa. Phylogenetic reconstruction suggests L10 could represent a missing link in the evolutionary and geographic migration histories of *M. africanum* (Guyeux et al., 2024).

Historically, *M. africanum* shows “intermediate” phenotypic characteristics between *M. tuberculosis* and *M. bovis* based on biochemical testing (Castets et al., 1968; Meyer and David, 1979; Thorel, 1980); however, lineage classification based on genotypic differences is more accurate than one based on phenotypic assays, also because phenotypic diversity among isolates of the same lineage or even sublineage cannot be excluded (Silva et al., 2022). Compared to *M. tuberculosis*, L5 and L6 are reported to have slower growth in culture, along with lower bacterial load and delayed disease progression (Cá et al., 2019; Baya et al., 2020; Balamurugan et al., 2022).

Phylogenetically, L5, L6, L9, L10, and the animal-adapted lineages share a common ancestor lacking the RD9, but L5 split from the common phylogenetic branch before the others. Some L5 genomes (L5.1.1) have also undergone RD711 deletion, while L6 has lost RD702 (Mostowy et al., 2004; De Jong et al., 2010; Ates et al., 2018; Coscolla et al., 2021; Comín et al., 2021). L6 has also undergone deletion of RD7, RD8, and RD10 regions (Gagneux, 2018; Comín et al., 2021; Balamurugan et al., 2022). L9 shares some genomic deletions with those of strains belonging to L6, such as RD702, but not with others, which are also present in the genomes of strains of animal-associated lineages, such as RD1 and RD5 (Coscolla et al., 2021; Silva et al., 2022). L10 lacks RD9, RD7, RD8, and RD10, and it harbors a specific large 9,134-no turning (nt) deletion (*Rv0613c*–*Rv0622*) in *M. tuberculosis* H37Rv (NC\_000962.3:706602–715,736) not observed in any other lineage (Guyeux et al., 2024).

Recently, whole-genome sequencing (WGS) analyses allowed the construction of detailed MTBC phylogenetic trees also based on several specific SNPs (Brites et al., 2018; Ates et al., 2018; Otchere et al., 2018; Sanoussi et al., 2021; Coscolla et al., 2021; Balamurugan et al., 2022). WGS has now become a fundamental tool for resolving epidemiological relationships, phylogeny, host adaptation, resistance and virulence determinants, etc. (Coscolla et al., 2021; Balamurugan et al., 2022). Phylogenetically, L5 is placed closer to the human-adapted MTBC and L6 closer to the animal-adapted strains (Gagneux, 2018). The proposed evolutionary scenario is that the L6 ancestor was a generalist pathogen that subsequently adapted to different host species, with the possible hypothesis that L6 strains may have originated from an animal reservoir (Silva et al., 2022). L6 also shows a more differentiated population structure than L5, with three distinct monophyletic main sublineages (L6.1, L6.2, and L6.3) that can be further subdivided into at least three other subgroups/sublineages each (Coscolla et al., 2021; Balamurugan et al., 2022).

The objectives of the present study were: to describe the tuberculosis case that occurred in a domestic cat and its etiology; to perform an in-depth genomics characterization by WGS and bioinformatic analysis of the isolate for identification at the lineage/subspecies level, and to compare it with other genomes available in public repositories, gaining an insight into phylogenetic aspects.

## 2 Materials and methods

### 2.1 Cat origin and clinical picture

In February 2023, a skin biopsy was taken from a 3-year-old spayed female domestic European Shorthair cat with multifocal nodular cutaneous lesions and respiratory problems, and was sent to our Institute for diagnostic purposes. The animal was an indoor cat kept in Rome, reportedly taken in as a stray kitten at a village located on the Ionian coast of southern Italy, Calabria region (Cropani municipality, Catanzaro province).

Reportedly, 7 months before sampling, the cat started coughing, and after 3 months, cutaneous nodules appeared on the end of the anterior paws. Simultaneously, palpable lymph nodes became enlarged, and the cat showed inappetence and depression. The cat was treated with prednisolone and antibiotics (fluoroquinolones and macrolides) for about 2 months, with improvement in the respiratory signs. In July 2023, the presence of cutaneous nodules increased and expanded to other sites, one on the tail became ulcerated. In April 2024, a computed tomography scan (CT scan) was performed, revealing several nodular neoformations at the head and muzzle level, tail, and limbs, with involvement of the afferent lymph nodes. The lung parenchyma presented a severe picture characterized by thickening of the bronchial network and widespread areas of hepatization.

The cat died in May 2024, but unfortunately, the owner did not request a necropsy, and the only sample received by our Institution was the skin biopsy collected in February 2023.

### 2.2 Skin biopsy, histopathological, and microbiological investigation

Histological sections of the biopsy were routinely processed and stained with hematoxylin and eosin (HE). Histochemical Ziehl–Neelsen (ZN) stain was also performed on five new different sections.

For bacteria isolation and identification, the tissue was cultured on Columbia Agar supplemented with 5% sheep blood (VWR, Belgium) and brain heart infusion broth; following incubation for up to a week under aerobic and microaerobic (10% CO_2_) conditions at 37 °C, growth colonies were subcultured and pure colonies screened using standard techniques including colony morphology, Gram staining, catalase test, oxidase test, and biochemically identified at species level with API test kits (bioMérieux, France). The biopsy was also cultured using specific solid commercial media for the isolation of *Mycobacterium* spp. (Stonebrink and Loewenstein-Jensen media, Microbiol S.n.c., Italy), following incubation under aerobic conditions at 37 °C and 42 °C. *Mycobacterium* solid media were periodically evaluated for bacterial growth for up to 3 months, following the guidelines outlined in the World Organization for Animal Health (WOAH) Manual of Diagnostic Tests and Vaccines for Terrestrial Animals 2022 (World Organisation for Animal Health, 2022).

### 2.3 *Mycobacterium* molecular identification and genomics

DNA from growth colonies referable to *Mycobacterium* spp. was extracted using the QIAamp DNA Mini Kit (Qiagen, Hilden, Germany) following the manufacturer’s protocol and as previously described (Iurescia et al., 2021). The extracted DNA was subjected to real-time polymerase chain reaction (PCR) (Ferrari et al., 2024) and a multiplex end-point PCR (Kulski et al., 1995) for the identification at the genus level and to assess their belonging to MTBC.

The MTBC isolates retrieved were also investigated by WGS analysis. Libraries for short-read pair-end sequencing were prepared using the Nextera XT DNA library preparation kit (Illumina, Inc., San Diego, CA, USA) following the Nextera XT R Guide 150319425031942 and sequenced on an Illumina platform (MiSeq). Quality trimming of the raw reads was performed using Trimmomatic version 0.39 with the following parameters: LEADING:30, TRAILING:30, SLIDINGWINDOW:10:20, MINLEN:50 (Bolger et al., 2014). Assembly was performed using SPAdes version 3.13.0 (Prjibelski et al., 2020). The quality of the assembly was addressed using QUAST version 5.0.2 (Gurevich et al., 2013).

Multilocus sequence typing (MLST) was performed using the scheme published in the pubMLST.org database (Jolley et al., 2018) by uploading the complete assembly. TB Profiler version 5.0.1 (Phelan et al., 2019), with its own database, was used for “*in silico*” ribotyping, assigning a lineage, and identifying the resistance and virulence genes. In particular, point mutations in chromosomal genes that confer antimicrobial resistance in the MTB complex (*M. tuberculosis*) were interpreted in accordance with the World Health Organization (WHO) catalogue of mutations in *M. tuberculosis* complex and their association with drug resistance (World Health Organization, 2023).

Resistance and virulence genes were confirmed using AMRFinderPlus version 3.12.8 (Feldgarden et al., 2021), with the following cut-offs: minimum 80% coverage and 80% identity.

For the identification of the regions of difference (RDs), our complete genome was mapped against the reference *M. tuberculosis* H37Rv (NC_000962.3) using minimap2 version 2.24-r1122 (Li, 2018), samtools version 1.12 (Danecek et al., 2021) for sorting and indexing, and IGV version 2.5.3 (Thorvaldsdóttir et al., 2013) for visualization.

For a first genetic identification, a tree including the following publicly available raw reads from different MTBC strains was built: ERR150046 (chimpanzee *bacillus*), SRR3745458 (dassie *bacillus*), ERR234255 and SRR998578 (*M. africanum*), SRR6705904 (*M. bovis*), DRR120409 (*M. caprae*), ERR027298 (*M. microti*), SRR3500411 (*M*. *mungi*), SRR5642712 (*M*. *orygis*), ERR970409 (*M*. *suricattae*), SRR1239339 (*M. pinnipedii*). For a more in-depth identification, our isolate was then compared with raw reads from 675 publicly available *M. africanum* L5, L6, and L9 strains and 5 related genomes that could not be classified into any of the known human- or animal-associated MTBC lineages.

For both analyses, SNP identification was performed by using Snippy version 4.6^1^ with the default parameters (minimum quality of the nucleotide set as 13; minimum coverage set as 10; minimum proportion of those reads that must differ from the reference set as 0.9), and using *M. tuberculosis* H37Rv as the reference strain. Duplicate genomes and isolates with >200.000 base pairs of the genome not aligned with the reference one were discarded.

The first MTBC phylogenetic tree was built using Randomized Axelerated Maximum Likelihood (RAxML) 8.2.12 (Stamatakis, 2014) and the maximum likelihood (ML) algorithm with the “gtrcat” model and 1,000 bootstrap inferences.

The second phylogenetic tree was constructed using FastTree version 2.1.11 (Price et al., 2010), using a general time-reversible model (gtr) (Supplementary Table S1). The visualization of the figures (trees) was created using iTol (Letunic and Bork, 2021).

The raw reads obtained were submitted to the European Nucleotide Archive (ENA) under the study accession number ERR14161356.

## 3 Results

### 3.1 Histopathological and microbiological investigation

**Figure 1:**
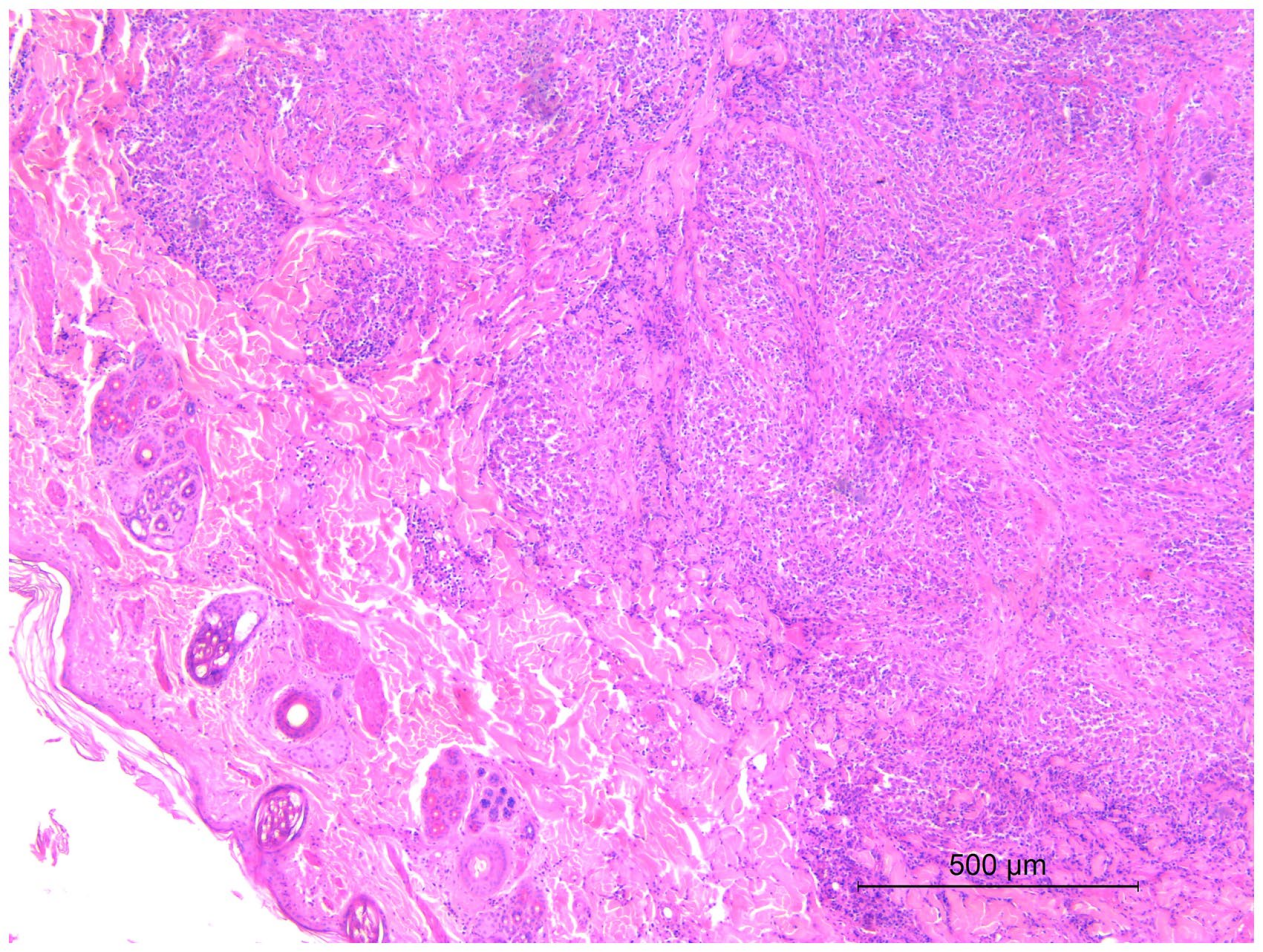
Histological section of a biopsy specimen collected from one of the cutaneous lesions. Non-capsulated multifocal to coalescent granulomas of variable size in the dermis. HE stain, 5×. Scale bar: 500 μm.

**Figure 2:**
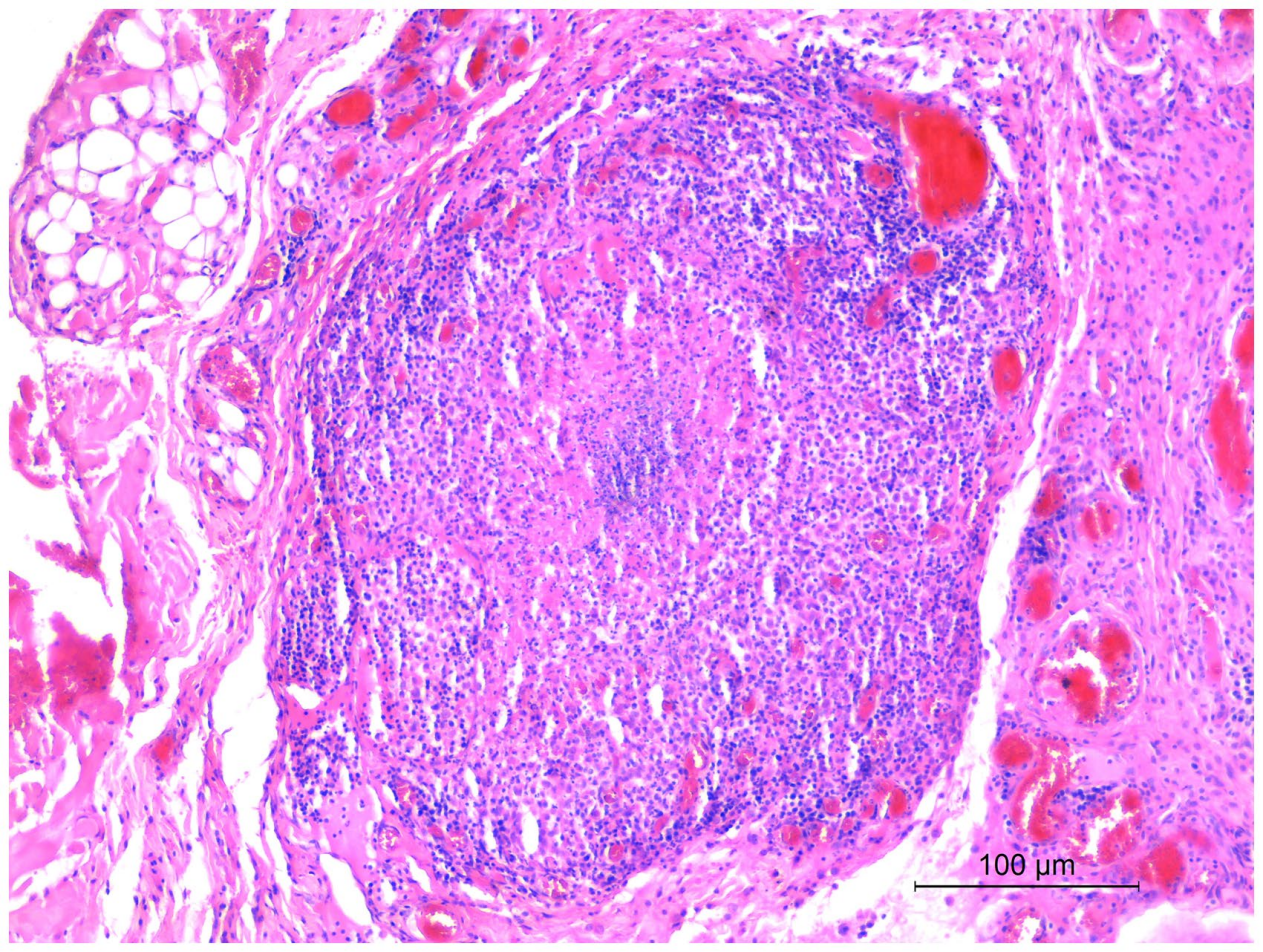
Histological section of a biopsy specimen collected from one of the cutaneous lesions. Smaller granulomas were characterized by a necrotic center with aggregates of degenerated neutrophils, and numerous macrophages with lymphocytic infiltrates at the periphery of the lesion. HE stain, 20×. Scale bar: 100 μm.

Microscopic examination of the skin lesions showed non-capsulated multifocal to coalescent granulomas of variable size in the dermis (Figure 1). Most of the large granulomas were composed of macrophages mixed with a few degenerated neutrophils and scattered lymphocytes, sometimes with multiple foci of necrosis. Smaller granulomas were characterized by a necrotic center with aggregates of degenerated neutrophils, and numerous macrophages with lymphocytic infiltrates at the periphery of the lesion (Figure 2). Multifocal areas of superficial skin ulceration were seen, which were associated with intradermal granulomas. Ziehl–Neelsen staining highlighted the presence of rare and scattered acid-fast bacilli, morphologically resembling mycobacteria, both in macrophages and in necrosis foci.

After approximately 12 weeks post-inoculation, suspect colonies referable to *Mycobacterium* spp. were detected on Loewenstein–Jensen media incubated at 37 °C. From the other bacterial standard cultures, *Staphylococus aureus* was isolated.

### 3.2 *Mycobacterium* molecular identification and genomics

*Mycobacterium* spp. suspected colonies from the skin biopsy were positive at the genus level and for MTBC by real-time PCR and multiplex end-point PCR, but negative for *M. bovis*, *M. caprae*, and *M. tuberculosis*. The WGS analysis using TBprofiler indicated that the isolate was *M. africanum* L6 (West-Africa 2). The octal spolygotype was 770777740000071. MLST analysis indicated that the isolate belonged to ST215 and to cgST-5847 (95% of loci matched). Moreover, the deletion of RD7, RD8, RD9, RD10, and RD702 was manually confirmed by mapping with the *M. tuberculosis* reference genome.

**Figure 3:**
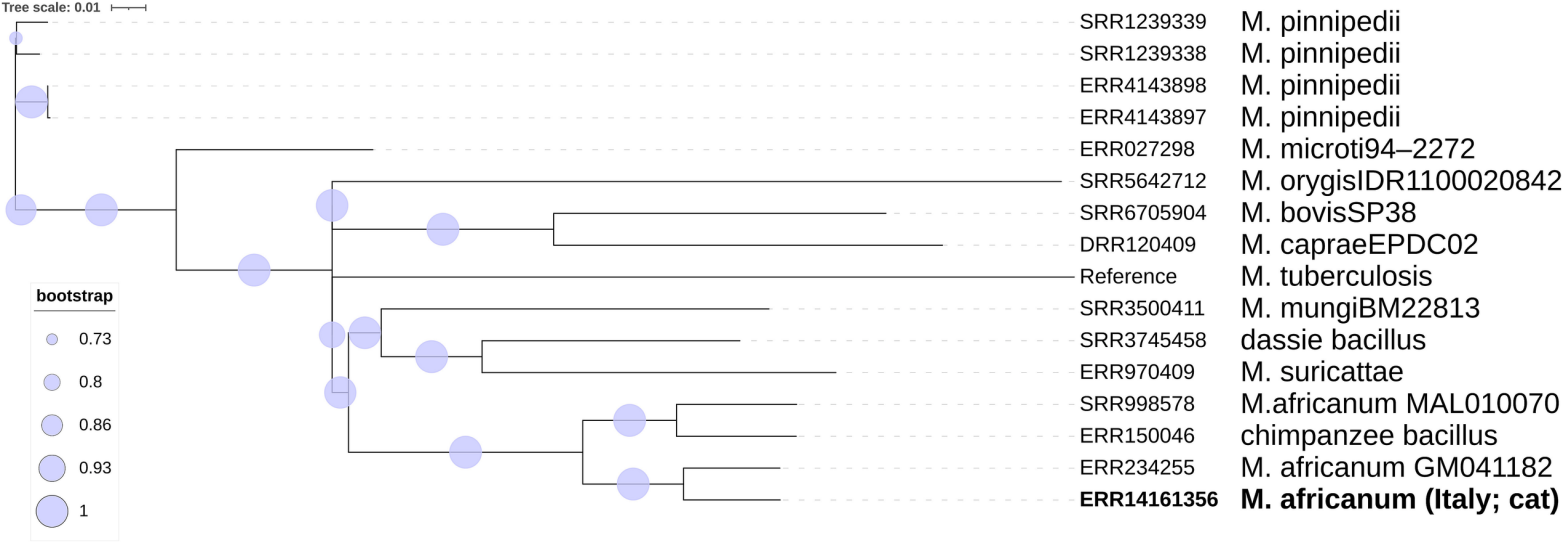
Phylogenetic SNP tree built with the ML algorithm of the main *Mycobacterium tuberculosis* variants.

**Figure 4:**
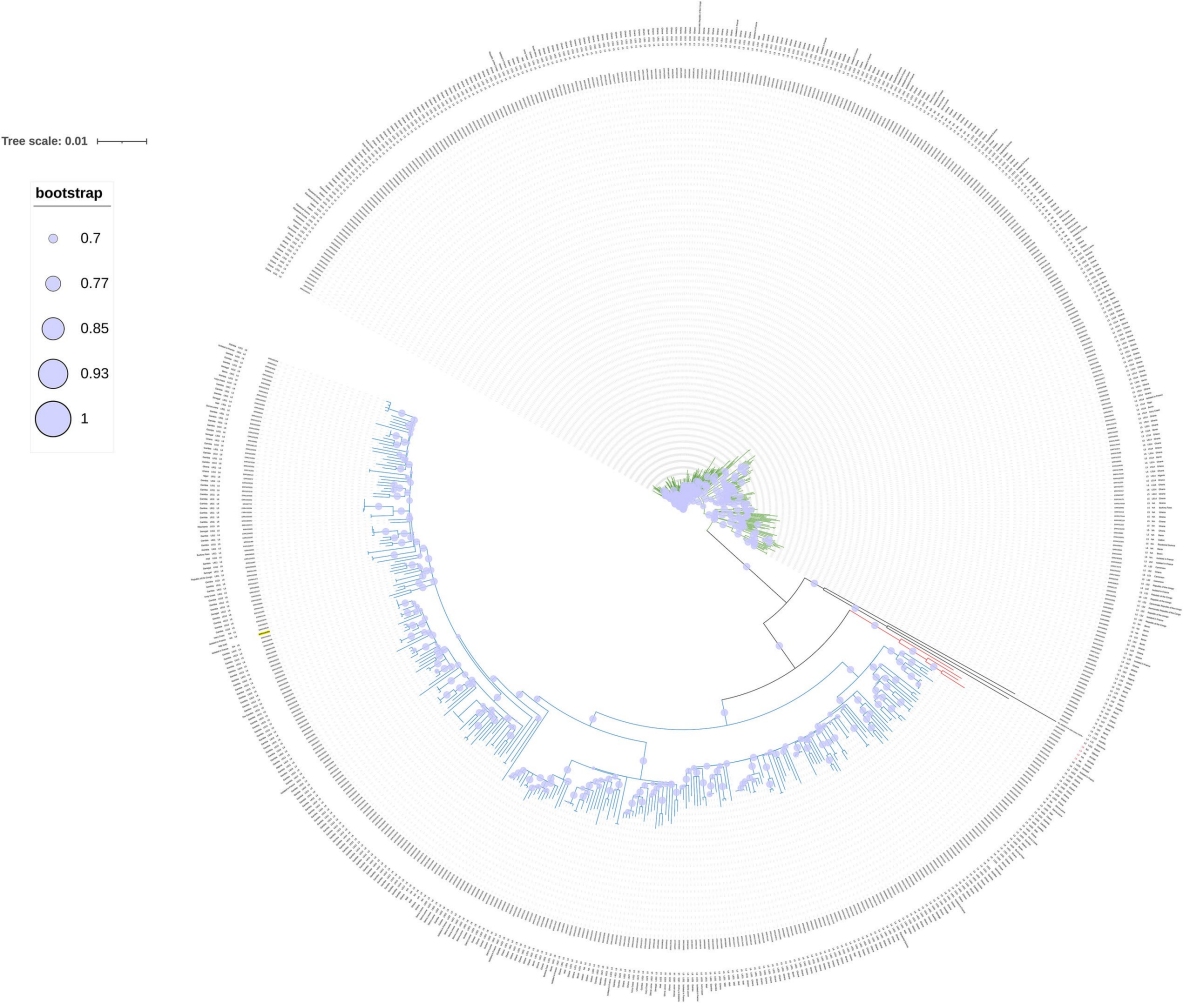
Circular phylogenetic SNPs tree built with 634 *M. africanum* genomes belonging to L5 (green), L6 (blue) and L9 (red), our cat isolate (ERR14161356, underlined in yellow) and the reference genome (NC_000962.3 *M. tuberculosis* H37Rv). Bootstrap values higher than 70% are represented with a circle within the clade.

SNP-based phylogenomic approach confirmed the identification as *M. africanum* (Figure 3) and included the genome of our isolate (ERR14161356) in a large cluster together with isolates mainly from Gambia and Ivory Coast, belonging to the L6.1.2 sublineage (Figure 4). According to this analysis, the most similar genome (229 SNPs) was a L6 (not specified sublineage) *M. africanum* isolated in France in 2007 from a human TB case (ERR2704811) (Ates et al., 2018).

The final assembly consisted of 153 contigs (≥1,000 bp) with an N50 of 77,339 bp and a total length of 4,606,140 bp, covering 98.21% of the genome with an average coverage depth of 58.65X. Using the AMRFinder tool, two natural (intrinsic) resistance genes encoding for macrolide and aminoglycoside, respectively, were found: *erm-37* and *aac*(2′)-Ic. The *bla*C gene, the *gene known to encode a beta-lactamase* in *M. tuberculosis*, was also detected. Using TBprofiler, some point mutations in specific genes, associated with resistance to rifampicin and rifapentine (*rpo*B), isoniazid (*inh*A and *kat*G), ethambutol (*emb*B and *emb*A), levofloxacin and moxifloxacin (*gyr*A and *gyr*B), bedaquiline and clofazimine (*atp*E), linezolid (*rpl*C), delamanid and pretomanid (*ddn*, *fbi*C, *fgd*1, and Rv2983), amikacin (*rrs*), streptomycin (*rrs* and *gid*), ethionamide and prothionamide (*eth*A and *inh*A) were found, but were classified as “of uncertain significance” or “Not associated with R” (Supplementary Table S1), according to the WHO guidelines (World Health Organization, 2023).

Regarding virulence genes, 139 genes already described in MTBC were detected, including *esx*A (6 kDa early secretory antigenic target), *esx*B (10 kDa culture filtrate antigen), the *mbt*A, *mbt*B, *mbt*C, *mbt*D, *mbt*E, *mbt*F, and *mbt*G genes, involved in the biosynthesis of the siderophore mycobactin and *plc*A, *plc*B, and *plc*C, encoding the phospholipase C exotoxin.

## 4 Discussion

We report, for the first time, an infection due to *M. africanum* in a domestic cat. The histopathological skin lesions observed resembled those of other cutaneous mycobacteriosis in cats (Malik et al., 2000; Lloret et al., 2013; Gunn-Moore, 2014; Sykes, 2025). Unfortunately, no further samples in addition to the skin nodules were made available for our laboratory, and the owner declined to perform a necropsy on the deceased animal. The lack of postmortem examination prevented the possibility of testing additional tissue samples to fully characterize the disease progression and systemic involvement. In any case, the reported clinical history and the computed tomography (CT) scan, with respiratory/pulmonary and lymph nodes involvement, were indicative of a generalized form consistent with a systemic TB case. The isolation of a *S. aureus* from the biopsy is probably attributable to a secondary infection that might have contributed to the cutaneous lesions.

In animals, *M. africanum* has been seldom reported in monkeys (Thorel, 1980; Coscolla et al., 2013), cattle (Weber et al., 1998; Rahim et al., 2007), pigs (Alfredsen and Saxegaard, 1992), and in hyrax (*Procavia capensis*) (Gudan et al., 2008). However, no animal reservoir has ever been postulated (Silva et al., 2022), and it is accepted that the epidemiology of infection basically relies on inter-human transmission. To the best of our knowledge, this represents the first report of *M. africanum* infection in a carnivore and in a companion animal. Recently, we have already witnessed zoonotic exposure of a *M. pinnipedii* from a captive sea lion (Alba et al., 2023). Indeed, the zoonotic risk of disease caused by MTBC from domestic or captive-bred carnivores should be taken into consideration (World Organisation for Animal Health, 2022; Gunn-Moore, 2014; Sykes, 2025; Rüfenacht et al., 2011; O’Halloran et al., 2024; Commandeur et al., 2025), and our findings highlight the need for awareness of potential MTBC transmission between species, including the potential role of humans as a reservoir. To the best of our knowledge, at the time of paper submission, no human TB cases in the cat household were reported.

The genomic investigation demonstrated that the implicated isolate was a *M. africanum* L6, and our isolate, together with an L6 isolated from a wild Chimpanzee in Côte d’Ivoire in 2013 (Coscolla et al., 2013), is at present the only fully genetically characterized *M. africanum* isolated from an animal.

This identification was further confirmed by the high similarity with other L6 genomes in the SNP-based phylogenomic approach, conducted as in the study by Coscolla et al. (2021), which placed the cat Italian isolate (ERR14161356) in a cluster together with isolates belonging to the L6.1.2 sublineage, a sublineage representing genomes originating in West Africa, mostly The Gambia (Coscolla et al., 2021). In particular, the closest genome to our isolate is a historical isolate detected in France (Ates et al., 2018). Unfortunately, no further information on the patient’s geographical origin is available about this French isolate, although a likely association with a West African exposure or origin has been suggested (R. Brosch, personal communication). TB caused by *M. africanum* strains is prevalent in West Africa, while outside it has been found mostly—but not only—in migrants from endemic areas (Silva et al., 2022). Indeed, inter-human transmission has also been reported in TB low-incidence European countries (Eldholm et al., 2021), where immigration and travel patterns from endemic areas facilitate local exposure. However, most of the sublineages within L6 have been reported to originate from West Africa, and only a few L6 strains were found in Central Africa or outside Africa (Coscolla et al., 2021). The cat was taken as a stray kitten at a camping site located on the Ionian coast of southern Italy, where in the nearby area are present two first migrant reception centers and several croplands, where workers coming from West Africa are often employed. Although the exact source of the infection in our case remains unknown, it might be speculated that the cat was initially exposed to infected humans/animals/fomites originally from West Africa, possibly including The Gambia. *M. africanum* geographical restriction, which contrasts with the widespread distribution of *M. tuberculosis* strains, remains largely unexplained. Regarding this, there are basically three main hypotheses: *M. africanum* is not able to compete with modern *M. tuberculosis* lineages; it is adapted to the African population, perhaps mediated through differential modulation of the immune response; and there could be an animal reservoir, making it a zoonotic disease (Comín et al., 2021; Silva et al., 2022). Furthermore, the true incidence of *M. africanum* infection may be underestimated whenever accurate laboratory differentiation within the MTBC isolates is not performed routinely on human cases (Comín et al., 2021). Based on human infections and experimental models, several Authors suggested that *M. africanum* strains and L6 may be less transmissible or virulent than *M. tuberculosis* strains (Sharma et al., 2016; Cá et al., 2019; Baya et al., 2020; Coscolla et al., 2021; Silva et al., 2022). Still, in our case, the cat became infected and likely died from TB. Some studies showed a high proportion of extrapulmonary TB caused by *M. africanum*, suggesting that these strains might show a different ability to cause pulmonary disease than *M. tuberculosis sensu stricto* (Ates et al., 2018; Comín et al., 2021). In our animal case, the cat likely had a generalized type of disease, with involvement of the skin and a clinical picture resembling those of other systemic mycobacteriosis in cats (Gunn-Moore, 2014).

The scientific interest in understanding the variation of *M. africanum* with respect to epidemiology and virulence is high (Yeboah-Manu et al., 2017; Gagneux, 2018; Ates et al., 2018; Coscolla et al., 2021; Balamurugan et al., 2022). Since the resistome and virulome found in our isolate are considered intrinsic/common features of *M. tuberculosis*, their presence in our cat *M. africanum* isolate could also be considered intrinsic and indicators of its zoonotic potential.

## 5 Conclusion

In conclusion, we report for the first time an infection due to *M. africanum* in a cat that most likely died from TB. Our molecular data show that the isolated strain belongs to L6, a lineage that has been considered closer (Silva et al., 2022) to certain animal-adapted lineages.

Although MTBC comprises host-adapted taxa, inter-species transmission between wild, captive, and domesticated animals and humans has been frequently reported, thanks to the plasticity of many of the MTBC taxa, posing a worldwide threat to both human and animal health (Vågene et al., 2022).

Indeed, the finding of tuberculosis caused by *M. africanum* in a carnivore further raises the possibility of the existence of non-human reservoirs, which may contribute to the main pattern of inter-human transmission in endemic areas, at least for some sublineages. TB-infected people who live in close contact with pets should be aware of the possibility of human-to-animal transmission, as proposed in our study. Conversely, citizens should be informed about the hazards posed by some well-known zoonotic lineages within the MTBC. In this regard, rapid and reliable etiological identification and characterization are of paramount importance, not only for an accurate diagnosis, but also for a correct approach to treatment and management options. Indeed, nowadays, the accuracy has greatly improved by genomics, which also allowed phylogenetic and phylogeographic insights.

In any case, suspected or confirmed tuberculosis cases in companion animals, in addition to an appropriate clinical case management, should also rapidly prompt prevention and control action within the household settings.

## Data Availability

The datasets presented in this study can be found in online repositories. The names of the repository/repositories and accession number(s) can be found inside the article.

## Footnotes

^1^ https://github.com/tseemann/snippy
